# Assessment of the Temporal and Spatial Variation of the Mercury Content of Lake Nokoué in Southern Benin Republic (West Africa)

**DOI:** 10.1155/2021/5412785

**Published:** 2021-10-05

**Authors:** Julien G. Adounkpe, N. B. Nadia Azon, Hermione W. Dégila, Peace Hounkpe, Robertson Amoussou

**Affiliations:** ^1^Laboratoire des Sciences et Techniques de l'Eau de l'Institut National de l'Eau, Université d'Abomey-Calavi, Benin; ^2^Laboratoire d'Ecologie Appliquée de la Faculté des Sciences Agronomiques, Université d'Abomey-Calavi, Benin; ^3^Laboratoire d'Etudes et de Surveillance Environnementale, Cotonou, Benin

## Abstract

Anthropogenic input of mercury into watersheds is becoming increasingly noticeable and is the cause of fishery products contamination. This had led to the Convention of Minamata signed and ratified by the vast majority of the countries in the world. Lake Nokoué in Benin Republic, the most fishery products provider in West Africa, is subject to mercury pollution. The health threat to both the benthic and the consumers of the fishery products from this lake has to be anticipated by precisely determining the dynamics of mercury contamination of the waters and sediments of Lake Nokoué, taking into account the anthropogenic contribution. Water and sediment samples were collected on 23 sites twice a month for four sampling campaigns. Parameters such as pH, salinity, COD, and SS are evaluated in the water. The assessment of total mercury is conducted by cold vapor atomic absorption according to the US EPA 7473 method, using a direct mercury analyzer (DMA-80). On average, the mercury content in Lake Nokoué water is 0.43 ± 0.57 *μ*g/kg. Its variation is linked not only to the seasonal variation but also to that of the suspended matter. South of the lake, the sediments are extremely polluted (5 ≤ Igeo) and present a risk of frequent effects for the benthic species present.

## 1. Introduction

Industrial, technological, and agricultural development is not without consequence on the environment [1]. Environmental pollutions, especially by heavy metals, are a threat to the aquatic ecosystems. Indeed, mercury pollution of surface water in Benin Republic is generally linked to agricultural activities and artisanal gold mining in northern Benin [2]. In the southern part of the country, the contamination of Lake Nokoué is strongly linked to the poor management of household and urban waste [3]. Lake Nokoué, being the largest lake in southern Benin, represents the base of the Ouémé river delta and communicates with watercourses such as the Ouémé river, the Djonou river, the Sô river, and the lagoon of Porto-Novo. The latter has been shown to be subject to mercury contamination [3].

**Figure 1:**
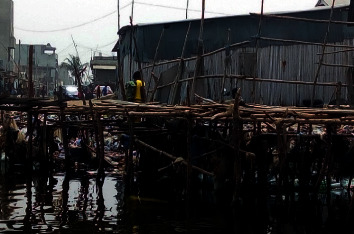
Human settlement on the Lake Nokoué bank at Yénawa-Cotonou (southeast of Lake Nokoué).

**Figure 2:**
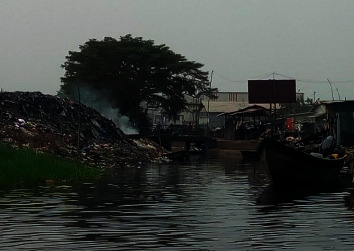
Wild dumpster at Vossa-Cotonou (southwest of Lake Nokoué).

Lake Nokoué hosts and is surrounded by several cities that use various types of batteries as their major sources of electricity. Those batteries are potential sources of mercury [1]. The banks of the lake are under the influence of large metropoles such as Cotonou, the economic capital of the country, and Abomey-Calavi, the dormitory city, where sanitation is of great concerns (Figures 1 and 2 ). Previous studies showed the presence of mercury in the flesh of fish species such as fish, shrimp, and oysters [4]. Even though if the current levels of mercury content seem not alarming, it should be noted that the bioamplifying power of the metal in the trophic network can rapidly degrade the current situation [5].

Additionally, mercury in the aquatic environment not only intoxicates the water body living organisms but can also be stored in the sediments where the bottom dwellers are exposed to its toxic effects. Later on, given certain atmospheric conditions, it can re-surface and get into the water body [6]. It is therefore of upmost importance to anticipate mercury pollution of the aquatic system in order to prevent its harmful consequences on aquatic fauna. This requires a quantification of mercury not only in the water but also and especially in the sediment for necessarily actions. The current study investigates the anthropogenic influence on the mercury pollution of the waters and sediments of Lake Nokoué, while taking into account the effect of seasonal variation.

## 2. Material and Method

### 2.1. Sampling

From July 2019 to January 2020, 23 sampling points were monitored bimonthly on the tributaries of Lake Nokoué, thus taking into account all the climatic seasons of the year. Thus, the junctions between Lake Nokoué and the other watercourses such as the Ouémé river, the Djonou river, the Sô river, and the Porto-Novo lagoon were sampled. Also the lake towns and the discharge points of the rainwater collection pipes were collected (Table 1).

Water samples were taken from a depth of 0.5 m below the water surface and transferred to 125 mL pretreated bottles. The bottles were protected from the air by putting them in plastic bags with zip, thus avoiding any atmospheric contamination of the samples stored with concentrated nitric acid (37%) at 5% v/v.

Sediment samples were also collected from each of the 23 sites using a Van Veen grab at a depth between 0 and 5 cm. All the samples were labeled and stored at a temperature between 2 and 8°C according to the standard NF EN ISO 5667-15, 2009.

### 2.2. Chemical Analysis

#### 2.2.1. Physicochemical Parameters

The water samples were subjected to measurements such as pH, salinity, conductivity in situ, and chemical oxygen demand (COD) and suspended solids (SS) in the laboratory. The parameters were measured in situ using a YSI multiparameter water quality meter from Fisher Scientific.

The COD was evaluated by titrimetry in accordance with the AFNOR T90-101 standard, and the SS were assessed following the AFNOR T90-105 standard.

#### 2.2.2. Determination of Mercury Content

Total mercury content was determined in both water and sediment samples using a direct mercury analyzer (DMA-80). The method has been extensively described elsewhere [1, 7, 8]. In short, this mercury analyzer has the particularity to directly assay the metal without any chemical pretreatment. It has a system of three ovens, the first of which is used for drying and decomposing the sample. The second furnace serves as a catalytic chamber, while the last furnace has a gold trap that captures the mercury released from the sample for a spectrometric reading. Thus, mercury is detected by cold vapor atomic absorption spectrometry by using the 7473 USPEA method.

### 2.3. Evaluation of Sediment Quality

The ecological quality evaluation of the sediment samples was made through four factors [9, 10]. The assessment of the geo-accumulation index (Igeo) is conducted according to the following formula:

(1) $$\begin{array}{c} \text{Igeo}=\log\;2\left(\frac{Cm}{1,5*Cfg}\right), \end{array}$$

where *Cm* corresponds to the measured mercury concentration, *Cfg* corresponds to the geological concentration of mercury, and 1.5 is the correction factor for the geochemical background [11, 12].

Taking into account the mercury ratio in the sediment without considering the heterogeneity of the sediments is appreciated by the contamination factor [13], and the formula is

(2) $$\begin{array}{c} \text{FC}=\left(\frac{Cm}{Cfg}\right). \end{array}$$

Finally, the threshold effect level (TEL) and the probable effect level (PEL) made it possible to assess the ecological quality of the sediments studied [14].

### 2.4. Statistical Analysis of Data

Following the descriptive analysis which is carried out on all the data, the distribution of mercury in the water and sediment matrices is assessed by the box-whisker plot. Analysis of variances is performed to assess the effect of sampling areas and other parameters on the variation in mercury content.

## 3. Results and Discussion

### 3.1. Lake Nokoué Water Physicochemical Parameters of Distribution

Table 2 presents the descriptive statistics of all the physicochemical parameters measured in the water of Lake Nokoué. The water temperature varies from 26.40°C to 31.70°C with an average of 29.37°C. This temperature amplitude is included in the range of temperature variation (24 to 35°C) that favors good fish growth in lagoon waters [15]. It is therefore understandable that Lake Nokoué be a very good fishery products sustainer.

**Figure 3:**
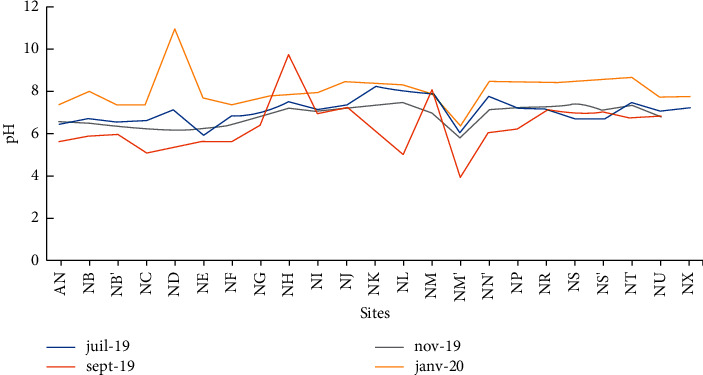
Temporal variation of the pH in the water.

The pH of the water in Lake Nokoué varies between 3.90 and 11.18 with an average of 7.10 (Table 2). This average value (pH = 7.2) corroborates observations by Djihouessi in 2018 related the waters of Lake Nokoué for the same period of the year [16]. The low pH value observed at Lake Nokoué could be due to occasional organic pollution, linked to domestic and industrial discharges or the effect of palm tree leaves and other plants used as fish habitat, the so-called acadjas [17]. In addition, the lowest pH values recorded during the month of September corresponding to the short rainy season are 4.96 and 3.9, respectively, at the Djonou river tributary (NM) and the point discharge of the rainwater draining pipe at Vossa (NM′). This may be related to the nature of the ions dragged by the tributary or the pipeline into the lake (Figure 3). The pH values in particular during the month of January, period of water recession and dry season where the highest pH values are recorded, could mean that the seasonal variation or the water height influences the acidic or basic character of the water of Lake Nokoué. This same observation is made by Buhungu et al. in 2018 about the Kinyankonge fishpond in Burundi [18].

The conductivity of the water in Lake Nokoué water varies greatly with a minimum of 1 *μ*s/cm, a maximum of 32723 *μ*s/cm, and an average of 4992.34 *μ*s/cm (Table 2). This average value greatly exceeds the limit value of 1000 *μ*s/cm set by the WHO for natural waters. However, the average value corroborates with the results found by Avmadi in 2019 (3482 *μ*s/cm) in Lake Togo. The high conductivity values obtained in the waters of Lake Nokoué could be explained by the variation of the tides on one hand and by the influence of human activities on the other hand. Indeed, the measurement of the conductivity of a water body, which is in itself the evaluation of the degree of ionization of this medium, depends essentially on the presence and the concentration of the various ions in the water [19]. However, given that Lake Nokoué is connected to the Atlantic Ocean via the channel of Cotonou (see map), the highly ionized seawater income greatly impacts the conductivity of the lake.

The salinity of Lake Nokoué varies from 0 ppt to 18.13 ppt with an average of 2.87 ppt in Lake Nokoué (Table 2). These results corroborate with the ones observed (0 ppt to 16.2 ppt) in Lake Nokoué by Djihouessi in 2018. Finally, the chemical oxygen demand varies between 91.43 mg/L and 3072 mg/L with an average of 337.96 mg/L in the waters of Lake Nokoué. These values are much higher than those obtained in the Chari River [20].

**Figure 4:**
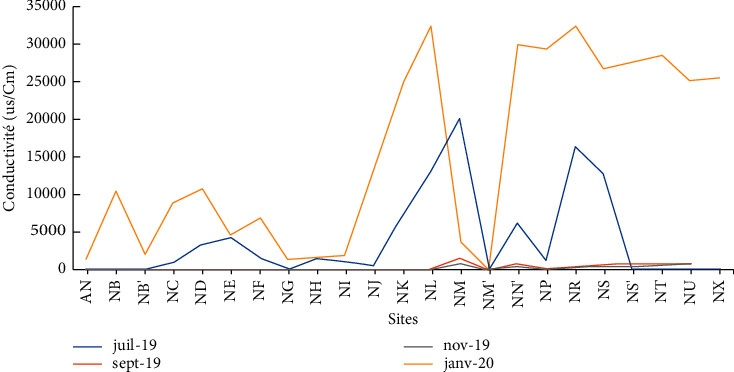
Temporal variation of the conductivity of Lake Nokoué.

**Figure 5:**
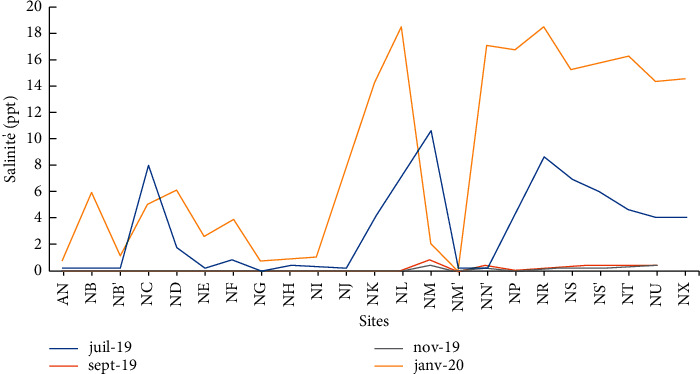
Temporal variation of the salinity in the water of Lake Nokoué.

The highest values of conductivity and salinity are observed during the months of July and January (Figures 4 and 5). These periods correspond, respectively, to the great rainy season and the great dry season. Indeed, during the rainy period, the runoff water, by washing off the soils, drains ionic charges into the lake. Hence, the high values observed during July with peaks of 20382.57 *μ*s/cm and 16403.57 *μ*s/cm for conductivity and peaks of 10.67 ppt and 8.80 ppt for salinity, respectively, at the discharge points of the rainwater pipes at Vossa (NM) and Adogléta (NR) are logical. During the periods of high-water level (August–September), the conductivity recorded is less than 100 *μ*s/cm and the salinity less than 1 ppt at all sampled sites. When the water recedes by January, the conductivity and salinity experience spectacular variations with conductivities over 20,000 *μ*s/cm and salinities over 10 ppt in the downstream area of the lake (Ladji, Vasso, Adogléta, Agbato, and Yénawa).

### 3.2. Mercury Content Variation in Water and Sediment

**Figure 6:**
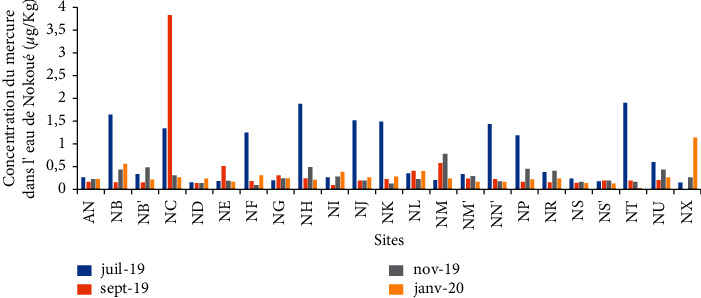
Variation in the concentration of mercury in the water of Lake Nokoué.

The mercury content in the waters of Lake Nokoué varies from 0.02 *μ*g/kg to 4.93 *μ*g/kg with an average of 0.43 ± 0.57 *μ*g/kg (Table 3). Although being remarkably present in all the sampling sites, the quantity of mercury during the month of July remains below 2 *μ*g/kg, the limit value for mercury in surface water according to Rodier et al. in 2009 [21] (Figure 6). However, the mercury concentrations in the tributaries of the Sô river (NF), the Porto-Novo lagoon (NH), the Ouémé river (NG), urban wastewater discharge pipes (NT, NP) and (NU), the Cotonou channel (NN′), and certain specific sites (NJ, NB) for the same month have an average concentration that exceeds 1 *μ*g/L that is obtained in the waters of the Konkouré river estuary [22]. 1 *μ*g/L also represents the limit value for mercury set by the WHO. This high mercury content may be due to the leaching from dumpsters alongside the lake. The low mercury contents (<1 *μ*g/L) are observed in September, November, and December where there is no much of leaching. The exceptional peak of 3.81 *μ*g/L of mercury obtained during the month of September in Ganvié (NC) can be explained by a point contamination, which would certainly be linked to the current activities of the populations of the city.

**Figure 7:**
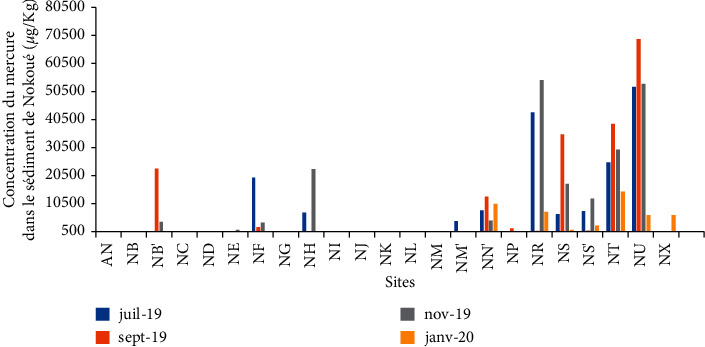
Variation in the content of mercury in the sediments of Lake Nokoué.

The mercury content in the sediments collected from the same sites as the water samples from July to January varies from 21.74 *μ*g/kg to 80976.68 *μ*g/kg with an average of 7139.02 *μ*g/kg (Figure 7). The highest mercury content in the sediment of Lake Nokoué is obtained at the tributary's outlets, at the discharge points of the pipes, and at the point of the lake's outlet into the channel of Cotonou. Each of these sediments has concentrations greater than 0.5 mg/kg of mercury, limit values according to the OSPAR standard [23, 24]. This is an additional proof that the probable sources of mercury contamination of Lake Nokoué are households, urban discharges, and wild dumpsters (NC, NT, NM, NS, NE, NF, etc.).

**Figure 8:**
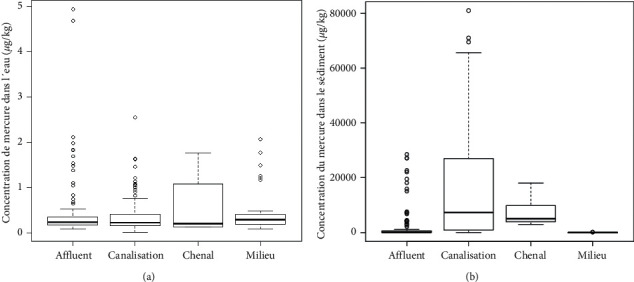
Global overview of the distribution of mercury in water (a) and sediment (b) by study area from July 2019 to January 2020.

It should be noted that the concentration of mercury presents a better distribution within the water matrix at the level of the tributaries and within the body of the lake itself (Figure 8(a)). However, at the pipe's outlet and at the inlet of the channel of Cotonou, the behavior of the metal can be linked to a possible swing of the metal contained in the water column toward the sedimentary medium. The high mercury contents are obtained at the level of the discharge points of the rainwater collection pipes into the lake (Figure 8(b)). Even if mercury does not seem to present a better distribution in the sedimentary environment at those areas, the 4th quartile exhibiting concentrations ranging from 25,000 to 60,000 *μ*g/kg and the 3rd quartile observations vary between approximately 8,000 *μ*g/kg and 25000 *μ*g/kg give the proof of anthropogenic pollution. This would be due not only to the leaching from the soil by runoff water but also to the household garbage often dumped in the water collectors by the population and to the presence of the refuse installed directly on the banks. Thus, it emerges that the mercurial concentration of the sedimentary environment of Lake Nokoué is strongly influenced by the nonmanagement of urban waste from the city of Cotonou.

### 3.3. Influence of the Physicochemical Parameters of Water on the Variation of Mercury

The ANOVA results (Table 4) reveal a significant difference between the campaign periods (*p* < 0.001). The variation of mercury in lake waters is strongly dependent on suspended solids with a probability less than 0.05. The effect of categorical interactions shows that the period of the campaigns influences the variation of mercury in water. Thus, the seasons or periods of flooding and falling considerably influence the mercury content in the waters of Nokoué. This confirms that during the rainy period, the water inflow into the lake contributes to a mercury supply in the body of water. In short, the season and the height of the water influence the mercury content in the water, which in itself remains dependent on suspended matter present in the medium.

In addition, the sediment sampling depth shows a significant difference (*p* < 0.001) with regard to the mercury content in the sedimentary environment. The mercury content does not seem to be influenced by the parameters of the water in any way (Table 5). However, the results of the effects of the interactions of the categorical variables reveal a level of significance at the threshold of *p* < 0.05. This confirms the influence of the sampling periods on the variation of the mercury content in the sedimentary environment. Thus, the mercury present in the sediment of Lake Nokoué depends on the seasons, the water level, and the areas sampled.

### 3.4. Influence of Human Activities on Mercury Pollution

**Figure 9:**
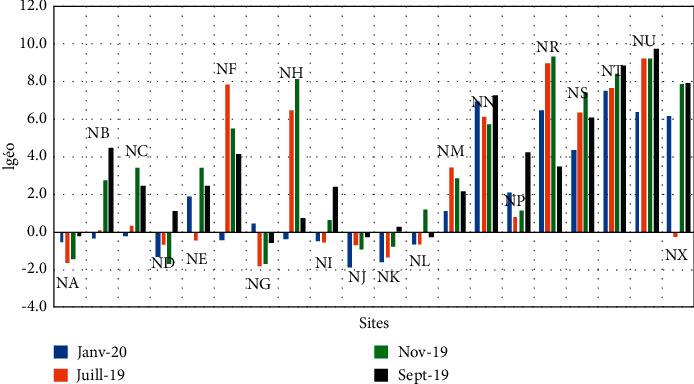
Averages of Igeo values of the sediments with respect to the seasons.

**Figure 10:**
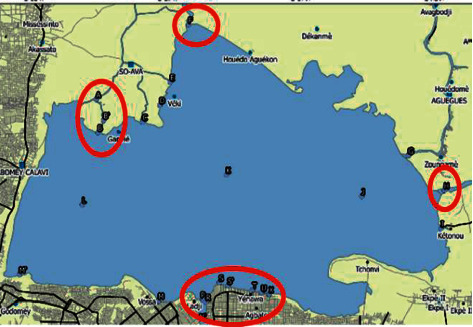
Visualization of sites with a high Igeo.

From the seasonal monitoring of Lake Nokoué sediments from July 2019 to January 2020 (Figure 9), it appears that all the sites located on the body of the lake itself (NL, NK, NJ) are free of pollution (Igeo < 0). The sites (NN, NS, NS′, NU, NT, NX) related to the rainwater collector discharge points are extremely polluted (5 ≤Igeo) and this was observed in the four sampling campaigns. The sediments collected at the entrance to the main tributary of the Sô (NF) and the entrance to the tributary of the Porto-Novo lagoon (NH) were found to be extremely polluted during the months of July and November 2019 and then heavily polluted at the NF site in September. In addition, the sediments collected in November and January in Ganvié (NB, NC) are slightly or moderately polluted. We deduce that the mercury quality of the lake is influenced by the domestic discharges, the temporary contribution of the Sô river and the Porto-Novo lagoon, and finally the permanent contribution of urban wastewater associated with refuse located on the shores of the lake (Figure 10).

**Figure 11:**
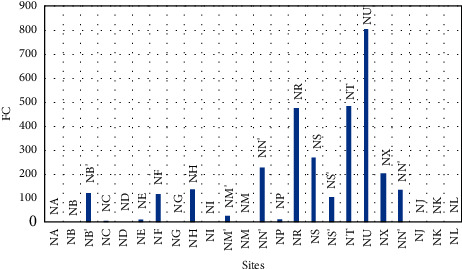
Average CF values for Lake Nokoué sediments 2019–2020.

The average FC value of the sediments collected between July 2019 and January 2020 on Lake Nokoué reveals a great variability in the mercury contamination of the sediments. It appears that the NA, NG, NJ, and NK sites are not contaminated or are slightly contaminated with mercury. ND and NK sites are moderately contaminated with mercury, and NI and NM sites are significantly contaminated. In accordance with the observation made with the Igeo, the sediments in connection with the stormwater discharge sites, the refuse, the main tributary of the Sô, the tributary of Djonou, and the Porto-Novo lagoon, of which the CF exceeds all Carballeira classifications [21], are highly contaminated with mercury with the highest FC of 805.35 obtained at the NU site (located at Yénawa) (Figure 11).

**Figure 12:**
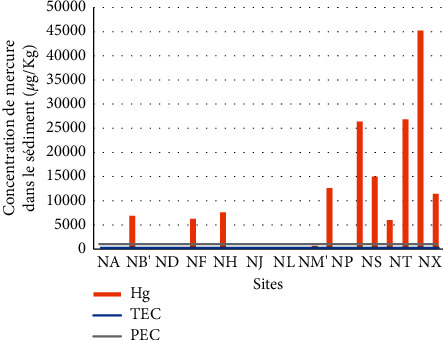
Mercury content in Nokoué sediments with regard to toxicity thresholds.

47.82% of the sites sampled on Lake Nokoué between July 2019 and January 2020 have mercury concentrations between the PEC and TEC values. This means that each of the sites presents a risk of occasional effects on benthic communities. All the remaining sites except the one at the entrance to the tributary of Djonou (NM′) show mercury concentrations that exceed the PEC threshold. This means that they are likely to cause frequent harms to the benthic fauna (Figure 12).

## 4. Conclusion

This study clearly demonstrates the seasonal variation and the spatial distribution of mercury content of Lake Nokoué, both in the water body and sediment. The water body of the lake receives mercury from nonpoint sources that were seemingly linked to cotton production fields runoff and channeled through the Ouémé river, the Sô river, and the Djonou river. The high mercury content at the outlets of urban runoff drainage pipes gives additional proofs of nonpoint sources of mercury pollution of the lake. However, the high mercury concentration in the sediment at some strategic points of the lake, such as near some wild dumpsters, lake cities such as Ganvié, Zouko, and Sô-Ava, and Cotonou can be linked to point sources of pollution. The study has demonstrated that the concentration of mercury contained in the water and the sediments is influenced by the lake's water level, the rainy or dry season. Thus, the evaluation of the ecological quality of the lake sediments reveals that the sediments at the entrance to the Djonou river present risks of occasional effects to the benthic fauna and that those taken at the entrance to the Sô, the lagoon of Porto-Novo, and those of Ganvié, as well as at all the rainwater discharge sites and refuse dumps are likely to have frequent effects to the benthic fauna. In view of these findings, the stakeholders of Lake Nokoué must be sensitized for the protection of this important water body to spare the aquatic lives.

## Figures and Tables

**Table 1:** Geographical coordinates of the sampled sites.

Sampled areas	Code	X coordinate	Y coordinate
Tributary of the Sô	NA	2.3878816667	6.4770066667
Tributary of the Sô	NB′	2.3911616667	6.4678444444
Tributary of the Sô	NB	2.3886111111	6.4624933333
Tributary of the Sô	NC	2.4087833333	6.4671116667
Tributary of the Sô	ND	2.4165116667	6.4745083333
Tributary of the Sô	NE	2.421208333	6.484571667
Tributary of the Sô	NF	2.4294266667	6.5094333333
Affluent of Aguégués	NG	2.5284233333	6.4524833333
Tributary to the lagoon of Porto-Novo	NH	2.5444433333	6.4373983333
Periphery	NI	2.5430066667	6.4208255555
Middle	NJ	2.5074966667	6.4348183333
Middle	NK	2.4464716667	6.4442216667
Middle	NL	2.3816066667	6.4311633333
Pipe rejection	NM	2.4158433333	6.3891211111
Tributary of the Djonou	NM′	2.3534783333	6.4006111111
Enter the Cotonou channel	NN′	2.3534783333	6.4006111111
Pipe rejection	NP	2.4359583333	6.3897016667
Pipe rejection	NR	2.4373444444	6.3886255555
Pipe rejection	NS	2.4432916667	6.3970316667
Pipe rejection	NS′	2.4471766667	6.3957355555
Pipe rejection	NT	2.4582999999	6.3938366667
Pipe rejection	NU	2.4623555555	6.3927016667
Pipe rejection	NX	2.4658155555	6.3919355555

**Table 2:** Statistical data of the physicochemical parameters of water.

Parameters	*n*	Mean	Median	Min	Max	Sd	se
Temp (°C)	**276**	**29.37**	**29.50**	**26.40**	**31.70**	**1.09**	**0.07**
pH	**276**	**7.10**	**7.13**	**3.90**	**11.18**	**1.02**	**0.06**
Cond (*μ*s/cm)	**276**	**4992.34**	**499.45**	**1.00**	**32723.00**	**9080.87**	**546.60**
Sal (ppt)	**276**	**2.87**	**0.24**	**0.00**	**18.13**	**4.93**	**0.30**
COD (mg/L)	**276**	**337.96**	**182.86**	**91.43**	**3072.00**	**468.58**	**28.20**
MES (mg/L)	**276**	**0.08**	**0.03**	**0.00**	**1.48**	**0.20**	**0.01**
Hg-Suf (*μ*g/L)	**276**	**0.43**	**0.24**	**0.02**	**4.93**	**0.57**	**0.03**
Hg-Sed (*μ*g/Kg)	**276**	**7139.02**	**218.42**	**21.74**	**80976.68**	**14449.48**	**869.76**

**Table 3:** Statistical data of the mercury concentration in water and sediment.

Parameters	*n*	Mean	Median	min	Max	Sd	se
Hg-water (*μ*g/L)	276	0.43	0.23	0.02	4.93	0.57	0.03
Hg-Sed (*μ*g/kg)	276	7139.02	218.42	21.74	80976.68	14449.48	869.76

**Table 4:** Analysis of variances between the parameters of Lake Nokoué water.

	DF	Sum sq	Mean sq	*F* value	Pr (>)
Date	3	10.23	3.409	11.627	3.52 e-07 ^*∗∗∗*^
Zone	3	0.55	0.183	0.624	0.6001
Temp	1	0.51	0.513	1.748	0.1873
pH	1	0.07	0.069	0.237	0.6268
DCO	1	0.02	0.021	0.072	0.7892
Sal	1	0.00	0.00	0.000	0.9985
MES	1	1.20	1.204	4.106	0.0438 ^*∗*^
Cond	1	0.30	0.299	1.020	0.3133

**Table 5:** ANOVA result between parameters and mercury in the sediment of Lake Nokoué.

	DF	Sum sq	Mean sq	*F* value	Pr (>)
Date	3	2.430e+09	8.099e +08	5.486	0.001138 ^*∗*^ ^*∗*^
Zone	3	1.342e +10	4.475e +09	30.314	<2e-16 ^*∗*^ ^*∗*^ ^*∗*^
Temp	1	6.843e +07	6.843e +07	0.464	0.496546
pH	1	2.504e+06	2.504e +06	0.017	0.896470
DCO	1	4.054e+06	4.054e+06	0.275	0.600671
Sal	1	3.664e+08	3.664e+08	2.482	0.116332
MES	1	3.259e+08	3.259e+08	2.208	0.138515
Cond	1	9.192e+06	9.192e+06	0.062	0.803141
Prof-sed	1	2.074e+09	2.074e+09	14.051	0.000219 ^*∗*^ ^*∗*^ ^*∗*^

## Data Availability

The data used to support the findings of this study are available within the article.
